# Phenolic Compounds of Red Wine *Aglianico del Vulture* Modulate the Functional Activity of Macrophages via Inhibition of *NF-κB* and the Citrate Pathway

**DOI:** 10.1155/2021/5533793

**Published:** 2021-05-25

**Authors:** Anna Santarsiero, Paolo Convertini, Antonio Vassallo, Valentina Santoro, Simona Todisco, Dominga Iacobazzi, Yvonne Fondufe-Mittendorf, Giuseppe Martelli, Marcos R. de Oliveira, Rosangela Montanaro, Vincenzo Brancaleone, Johannes Stöckl, Vittoria Infantino

**Affiliations:** ^1^Department of Science, University of Basilicata, Viale dell'Ateneo Lucano 10, 85100 Potenza, Italy; ^2^Department of Pharmacy, University of Salerno, Via Giovanni Paolo II 132, 84084 Salerno, Italy; ^3^Bristol Heart Institute, Bristol Medical School, University of Bristol, Bristol BS2 8HW, UK; ^4^Department of Molecular and Cellular Biochemistry, University of Kentucky, Lexington, KY 40536, USA; ^5^Departamento de Bioquímica Rua Ramiro Barcelos, Universidade Federal do Rio Grande do Sul (UFRGS), 2600 Anexo Santa Cecília, Porto Alegre, RS, Brazil; ^6^Institute of Immunology, Center for Pathophysiology, Infectiology and Immunology, Medical University of Vienna, 1090 Vienna, Austria

## Abstract

Phenolic compounds of red wine powder (RWP) extracted from the Italian red wine *Aglianico del Vulture* have been investigated for the potential immunomodulatory and anti-inflammatory capacity on human macrophages. These compounds reduce the secretion of IL-1*β*, IL-6, and TNF-*α* proinflammatory cytokines and increase the release of IL-10 anti-inflammatory cytokine induced by lipopolysaccharide (LPS). In addition, RWP restores Annexin A1 levels, thus involving activation of proresolutive pathways. Noteworthy, RWP lowers NF-*κ*B protein levels, promoter activity, and nuclear translocation. As a consequence of NF-*κ*B inhibition, reduced promoter activities of *SLC25A1*—encoding the mitochondrial citrate carrier (CIC)—and *ATP citrate lyase* (*ACLY*) metabolic genes have been observed. CIC, ACLY, and citrate are components of the citrate pathway: in LPS-activated macrophages, the mitochondrial citrate is exported by CIC into the cytosol where it is cleaved by ACLY in oxaloacetate and acetyl-CoA, precursors for ROS, NO^·^, and PGE_2_ inflammatory mediators. We identify the citrate pathway as a RWP target in carrying out its anti-inflammatory activity since RWP reduces CIC and ACLY protein levels, ACLY enzymatic activity, the cytosolic citrate concentration, and in turn ROS, NO^·^, PGE_2_, and histone acetylation levels. Overall findings suggest that RWP potentially restores macrophage homeostasis by suppressing inflammatory pathways and activating proresolutive processes.

## 1. Introduction

Immunomodulators are heterogeneous compounds capable to interact with the immune system to upregulate or downregulate specific biological aspects of the host response. For example, phenolic compounds scavenge free radicals, prevent lipid peroxidation, modulate inflammatory pathway, and block the secretion of proinflammatory cytokines [1]. Resveratrol counteracts the production of proinflammatory cytokines, while anthocyanidins downregulate the expression of cyclooxygenase 2 (COX2) in macrophages exposed to lipopolysaccharide (LPS) [2]. Interestingly, resveratrol is able to dampen inflammation and induce apoptosis in immune cells by triggering proresolutive mediator Annexin A1 (AnxA1) pathway [3, 4].

In addition, cyanidin-3-O-glucoside, petunidin-3-O-glucoside, and delphinidin-3-O-glucoside inhibit the master regulator of the immune function in mammalian cells, the transcription factor NF-*κ*B, in a mitogen-activated protein kinase- (MAPK-) dependent manner [5, 6]. Certain anthocyanidins can suppress the generation of reactive oxygen species (ROS) [7, 8]. For example, cyanidin-3-O-glucoside has great oxygen radical absorbance capacity (ORAC) *in vitro* [9] and delphinidin is one of the most active scavenger against superoxide anion [10]. Compounds found in red wine can also upregulate the transcription factor of the expression of antioxidant and detoxifying enzymes in mammalian cells, improving the cytoprotection against several types of stress [10].

In inflammatory processes, metabolic changes occur to meet the new energetic demands of cells. The result is the production of metabolites, which both can act as immune signaling molecules and supply substrates necessary for the biosynthesis of proinflammatory mediators [11, 12]. Activated dendritic cells and macrophages switch rapidly from a resting to an activated state characterized by a different metabolic profile. In particular, LPS or classically activated macrophages—also known as M1 macrophages—have high rates of glycolysis and pentose phosphate pathway while the Krebs cycle is broken at two points and fatty acid oxidation and oxidative phosphorylation are downregulated [11, 12]. The two breakpoints of Krebs cycle are at succinate dehydrogenase and isocitrate dehydrogenase, with consequent withdrawals of succinate and citrate from the cycle. Most of the citrate is channelled into the citrate pathway, made of the mitochondrial transporter citrate carrier (CIC) and the enzyme ATP citrate lyase (ACLY) [13–16]. CIC exports citrate from the mitochondria in exchange for malate. In the cytosol, ACLY cleaves it into oxaloacetate (OAA) and acetyl-coenzyme A (acetyl-CoA). OAA is converted to malate by cytosolic malate dehydrogenase 1 (MDH1) and to pyruvate by malic enzyme 1 (ME1) with consequent production of nicotinamide adenine dinucleotide phosphate (NADPH). Of note, both NADPH oxidase and inducible nitric oxide synthase (iNOS) need NADPH for ROS and nitric oxide (NO^·^) synthesis, respectively [13, 15]. Acetyl-CoA is processed into malonyl-coenzyme A (malonyl-CoA) by acetyl-coA carboxylase (ACC). Malonyl-CoA is a substrate for cholesterol or fatty acid synthesis. Therefore, it could be used for the production of arachidonic acid, a precursor for prostaglandin E_2_ (PGE_2_), a key modulator of inflammation with a crucial role in inflammatory diseases [13, 15]. Acetyl-CoA is also a substrate for protein and histone acetylation [17]. Moreover, citrate is implied in itaconate synthesis that modulates the production of different inflammatory mediators, acting as a negative regulator of inflammation [18]. The citrate pathway has a key role also in diseases such as Down syndrome and Behçet syndrome [19, 20].

The red wines have become popular in recent years due to their content of phenolic compounds with antioxidant activity as well as hypolipidemic and anti-inflammatory effects. Although different studies have been performed on the red wine compounds, most of them are aimed at investigating the chemical composition, the biodiversity, the genetic diversity, the pedigree reconstitution, and the general antioxidant properties. In this study, we investigated on the beneficial immunomodulatory effect of a powder rich in phenolic compounds from the red wine *Aglianico del Vulture*. It is one of the best Italian red wines that has never been studied. We have focused on the secretion of proinflammatory and anti-inflammatory cytokines, NF-*κ*B expression, the citrate pathway, and epigenetic modifications in LPS-activated human macrophages. Such studies will help to identify the targets for RWP and the development of potential therapeutics in the prevention and treatment of inflammatory chronic diseases.

## 2. Material and Methods

### 2.1. Wine Samples

Red wine (*Vitis vinifera L*., Aglianico cultivar) was provided by Cantine del Notaio (Rionero, Italy). Grapes were harvested in September 2018, samples were collected after grape pressing, and the wine fermentation was completed (no residual sugar was present into the wine). The samples were frozen and stored at -20°C before freeze drying. The wine samples (500 mL) in a glass cylinder were connected to a freeze drying apparatus and freeze-dried under vacuum using a Stellar Millrock ST8S5-l lyophilizer (Millrock Technology, Kingston, NY, USA).

### 2.2. LC–MS and LC–MS/MS Analyses

Part of whole and dealcoholized wine sample was dissolved in 100 *μ*L of 40% MeOH with 0.1% (*v*/*v*) formic acid at a concentration of 10 mg/mL and centrifuged (5 min, 13,000 rpm), and 1 *μ*L aliquots were injected in a UPLC–ESI–Qtrap system. Mass spectrometry-based analyses were carried out to evaluate the amount of specialized metabolites (delphinidin, cyanidin, and malvidin, all glucoside, caffeic and coumaric acids, resveratrol, and quercetin). Quali-quantitative analysis was carried out using an API6500 Q-Trap spectrometer (AB Sciex, Foster City, CA, USA) coupled with a Nexerax2 UHPLC apparatus (Shimadzu, Kyoto, Japan), working in both positive and negative MRM modes.

The instrumental parameters were optimized directly injecting solutions containing pure compounds. A Kinetex column (Phenomenex) (C18 100 Å, 50 mm × 2.6 *μ*m × 2.1 mm) was adopted for chromatographic analyses, and compounds were separated using a linear gradient from 5% to 50% of acetonitrile (eluent B) and water containing 0.1% formic acid (eluent A) over 5 minutes followed by a faster gradient until to 95% of B. The flow rate was 0.35 mL/min, and the injection volume was 1 *μ*L. To perform accurate quantitative analyses, 8 points (in the range 0.010–10 *μ*g/mL) calibration curves were built for all the standard compounds. The mean values ± standard deviation from at least three experiments were reported. All data were processed using Analyst software (ABSciex), and identification of compounds was based on retention times, accurate mass measurements, MS/MS data, exploration of specific spectral libraries and public repositories for MS-based metabolomic analysis [21], and comparison with data reported in the literature [7, 22–25].

### 2.3. Isolation of Human Monocytes from Whole Blood

Primary human monocytes were isolated from healthy donors after obtaining written informed consent. The study was performed in agreement with the Declaration of Helsinki and in accordance with the Committee on Human Research approved procedures. Venous blood was collected into K2 EDTA-coated BD vacutainer tubes (Becton, Dickinson and Company, Franklin Lakes, NJ, USA). Peripheral blood mononuclear cells (PBMCs) were separated by Histopaque-1077 (Sigma-Aldrich, St Louis, MO) density gradient centrifugation: whole blood was mixed with Hanks' Balanced Salt solution (HBSS, Sigma-Aldrich) at a ratio of 1 : 2 (*v*/*v*), layered on the top of Histopaque-1077 (Sigma-Aldrich) and centrifuged at 1000 x g for 15 minutes. The layer of mononuclear cells (PBMCs) at the interphase was recovered and washed twice in HBSS. PBMCs were incubated with CD14 antibody conjugated to magnetic beads (MACS®, Miltenyi Biotec GmbH, Bergisch Gladbach, Germany) for 15 minutes at 4°C. After washing, cells were loaded onto MACS® column (Miltenyi Biotec GmbH) placed in a magnetic field, and CD14- positive (CD14^+^) and CD14-negative (CD14^−^) populations were divided. The CD14^+^ monocytes were differentiated to macrophages by using 100 ng/mL human M-CSF in Roswell Park Memorial Institute (RPMI) 1640 medium (Thermo Fisher Scientific, San Jose, CA, USA) supplemented with 10% fetal bovine serum, 2 mM L-glutamine, 100 U/mL penicillin, and 100 *μ*g/mL streptomycin at 37°C in a humidified atmosphere of 5% CO_2_.

### 2.4. Cell Culture and Treatments

Human embryonic kidney 293 cells (HEK293, Sigma-Aldrich) were grown in Dulbecco's Modified Eagle Medium (DMEM, Thermo Fisher Scientific) supplemented with 10% fetal bovine serum, 2 mM L-glutamine, 100 U/mL penicillin, and 100 *μ*g/mL streptomycin in a humidified chamber with 5% CO_2_ at 37°C. To evaluate the immunomodulatory and anti-inflammatory properties of *Aglianico del Vulture* red wine, primary human monocytes and HEK293 cells were treated with RWP 20 or 200 *μ*g/mL for 1 hour. Then, inflammation was induced by 1 *μ*g/mL of lipopolysaccharide isolated and purified from *E. coli* strain EH100 (AdipoGen Life Sciences, Inc., San Diego, USA). Except for cytokines and PGE_2_ quantification, cells were washed twice with PBS at the end of LPS treatment before proceeding with subsequent analyses, as detailed further in the sections below.

### 2.5. Cell Count

CD14^+^ monocytes were seeded into a 96-well plate (2 × 10^4^ cells/well) and treated with a wide range of RWP concentrations: 2.5, 5, 10, 20, 50, 100, 200, 400, 800, 1600, and 3200 *μ*g/mL. After 72 hours, cell count was carried out by using the automated handheld Scepter 2.0 Cell Counter (Merck Millipore, Switzerland).

### 2.6. Quantification of Cytokines

CD14^+^ monocytes (5 × 10^5^ cells) were pretreated in 24-well plates with RWP 20 or 200 *μ*g/mL for 1 hour and then stimulated with 1 *μ*g/mL of LPS. Twenty-four hours later, cell-free supernatants were collected and assayed for the concentration of interleukins 1*β*, 6, and 10 (IL-1*β*, IL-6, and IL-10) and tumor necrosis factor *α* (TNF-*α*) by Luminex100 System (R&D Systems, Inc., Minneapolis, MN, USA) using specific matched-pair antibodies and recombinant cytokines as standards following the manufacturer's recommendations.

### 2.7. Western Blotting

Cellular pellet was resuspended in Laemmli buffer and boiled for 5 minutes at 100°C. Thirty micrograms of proteins were subjected to SDS-PAGE and then electroblotted onto nitrocellulose membranes. The membranes were blocked for 1 hour in a tris-buffered saline (TBS) solution containing 5% nonfat dry milk and 0.5% Tween 20 and then immunostained at 4°C overnight with anti-NF-*κ*B/p65 (ab7970, Abcam, Cambridge, MA), anti-CIC [26, 27], anti-ATP citrate lyase (ab157098, Abcam), anti-acetylated H3 (ab47915, Abcam), anti-total H3 (ab1791, Abcam), anti-AnxA1 (GTX101070, GeneTex), and anti-FPR2 or anti-*β*-actin (ab8227, Abcam) antibodies. Following 1-hour incubation with HRP Goat anti-Rabbit IgG secondary antibody (Santa Cruz Biotechnology, Santa Cruz, CA, USA), the immunoreactions were detected by using the horseradish peroxidase substrate WesternBright™ ECL (Advansta, Menlo Park, CA, USA) at Chemidoc™ XRS detection system equipped with Image Lab Software for image acquisition and densitometric analysis (Bio-Rad Laboratories, Hercules, CA, USA).

### 2.8. Transient Transfection

For monitoring the activity of the NF-*κ*B signaling pathway, HEK293 cells were transiently transfected with a NF-*κ*B reporter plasmid containing a firefly luciferase gene driven by five copies of NF-*κ*B response element (5′-GGGACTTTCC-3′) located upstream of the minimal TATA box promoter (pGL3–5xNF-*κ*B). To measure SLC25A1 gene promoter activity, HEK293 cells were transfected as previously described [28] using pGL3 basic-LUC vector (Promega, Madison, WI, USA) containing the −1785/−20 bp region of the SLC25A1 gene promoter (SLC25A1pGL3) upstream of the luciferase reporter gene [29]. For ACLY gene promoter activity, in pGL3, basic-LUC vector was cloned the −3116/−20 bp region of the ACLY gene promoter (called “3000”) or a deletion fragment of this region (called “1000”) [30]. To normalize the extent of transfection, cells were transfected with 10 ng of pRL-CMV (Promega). Twenty-four hours after transfection, HEK293 cells were triggered with LPS in the presence or absence of RWP 20 or 200 *μ*g/mL. The day after, cells were lysed and assayed for LUC activity by using the Dual-Luciferase® Reporter Assay System (Promega), according to the manufacturer's protocol.

### 2.9. Immunocytochemistry

Cells were induced with LPS for 1 or 3 hours in the presence or not of RWP 20 or 200 *μ*g/mL, then were washed in PBS and fixed by cross-linking with 3% paraformaldehyde solution. Following permeabilization with PBS +0.25% Triton X-100 (PBST) and blocking with PBST +1% BSA (bovine serum albumin), cells were incubated with anti-NF-*κ*B/p65 (ab7970, Abcam) primary antibody at 4°C overnight. The day after, Alexa Fluor 488 (Thermo Fisher Scientific) was used as a secondary antibody while Fluoroshield Mounting Medium with DAPI (ab104139, Abcam) was employed to preserve fluorescence and as a counterstain for DNA. The images were obtained with a fluorescence microscope (EVOS FLoid Cell Imaging Station, Thermo Fisher Scientific).

### 2.10. Quantification of Citrate

The amount of citrate was quantified in macrophages treated with LPS in the presence or not of RWP 20 or 200 *μ*g/mL by a fluorometric method using the Citrate Colorimetric/Fluorometric Assay Kit (BioVision, Milpitas, CA, USA) as per the manufacturer's instructions.

### 2.11. ACLY Activity

Primary monocytes were pretreated with RWP 20 or 200 *μ*g/mL for 1 hour and then activated to macrophages with LPS. At the end of treatments, cells were washed twice in ice-cold PBS. The cell pellet was resuspended in ice-cold 0.1% NP40 in PBS, and three freeze-melt cycles (-80°C for 8 minutes/40°C for 4 minutes) were performed. After centrifugation, supernatant was collected and protein concentration was determined by Bradford assay. ACLY activity was assessed by the coupled malic dehydrogenase method [31, 32], as previously described [30]. The specific ACLY activity was expressed as a percentage of the control after normalization to the protein concentration.

### 2.12. ROS, NO^·^, and PGE_2_ Detection

To evaluate ROS and NO^·^ levels, CD14^+^ monocytes were triggered by LPS in the presence or not of RWP 20 or 200 *μ*g/mL. Where indicated, cells were treated also with 5 mM sodium malate (Sigma-Aldrich) or 500 *μ*M NADPH (Sigma-Aldrich). Following 24 hours, ROS and NO^·^ concentrations were measured by using 6-Carboxy-2′,7′-Dichlorodihydrofluorescein Diacetate (DCF-DA, Thermo Fisher Scientific) and 4-Amino-5-Methylamino-2′,7′-Difluorofluorescein Diacetate (DAF-FM Diacetate, Thermo Fisher Scientific), respectively, as previously reported [33].

For PGE_2_ quantification, cells were exposed to RWP 20 or 200 *μ*g/mL for 1 hour and, where indicated, cotreated with 5 mM sodium acetate (Sigma-Aldrich); then inflammation was induced by LPS. At the end of 48 hours LPS treatment; PGE_2_ was measured by using DetectX® Prostaglandin E2 High Sensitivity Immunoassay Kit (Arbor Assays, Ann Arbor, MI, USA) as previously described [33].

### 2.13. Statistical Analysis

Results are shown as the means ± SD of, at least, three independent experiments. Statistical significance of differences was determined by using *one-way ANOVA* followed by *Dunnett's* or *Tukey's* tests for multiple comparisons. The statistical methods used for each experiment are detailed in figure legends. Asterisks in figures denote statistical significance: ^∗^*p* < 0.05, ^∗∗^*p* < 0.01, and ^∗∗∗^*p* < 0.001. When *Tukey's* post hoc test was performed, different letters indicate significant differences between treatments at *p* < 0.05.

## 3. Results

### 3.1. Composition and Identification of the Red Wine Powder Components

The red wine powder from *Aglianico del Vulture* was freeze-dried under vacuum. Quali-quantitative analysis of RWP was performed by LC-ESI-QTrap-MS/MS and LC-ESI-QTrap-MS analyses. The use of the Kinetex column and LC-ESI-MS/MS (alternating positive and negative ionization modes) allowed for the simultaneous separation and identification of all compounds. Compounds identified showed very good results in the optimized chromatographic column with retention times that ranged from 1.21 to 2.51 min. Extracted ion chromatograms for each compound are presented in Figure S1. Individual components were identified by comparison of their *m*/*z* values in the total ion current (TIC) profile with those of the selected compounds described in the literature. In particular, seven compounds were identified in RWP (Table 1) belonging to a wide variety of structurally different metabolic classes: phenolic acids (caffeic acid and p-coumaric acid); stilbenes (resveratrol); anthocyanidins (delphinidin-3-O-glucoside, cyanidin-3-O-glucoside, and malvidin 3-O-glucoside); and flavonols (quercetin).

The tested *Aglianico del Vulture* red wine powder contained significant amounts of anthocyanidins. In particular malvidin-3-*O*-glucoside was the most abundant (14.00 ± 0.23 mg/100 mL), followed by cyanidin-3-*O*-glucoside (1.30 ± 0.218 mg/100 mL) and delphinidin-3-*O*-glucoside (0.072 ± 0.003 mg/100 mL) (Table 1). Our results are in accordance with the typical anthocyanin profiling of *Aglianico* wine, in which malvidin 3-*O*-glucoside represents about 60% of total anthocyanidins while contents of cyanidin-3-*O*-glucoside and delphinidin-3-*O*-glucoside are very low (around 5%) [34]. The content of resveratrol in *Aglianico del Vulture* was 0.053 ± 0.01 mg/100 mL, similar to other Italian red wines [26, 35, 36]. Concentrations of caffeic acid and *p-*coumaric acid were 0.218 ± 0.047 mg/100 mL and 0.078 ± 0.002 mg/100 mL, respectively. Quercetin was determined equal to 0.785 ± 0.02 mg/100 mL as listed on Table 1.

### 3.2. Evaluation of RWP Toxicity on Primary Human Monocytes

**Figure 1:**
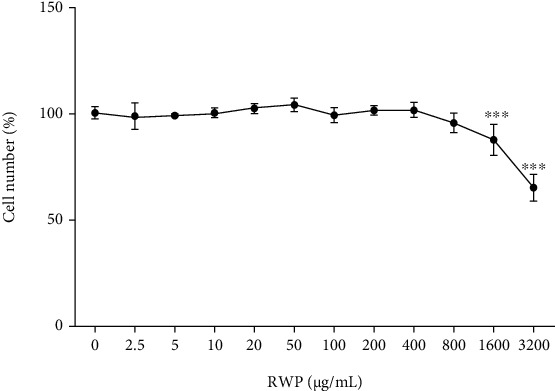
Effect of RWP on primary human monocyte cell number. Primary human monocytes were treated with increasing concentrations of RWP, ranging from 2.5 to 3200 *μ*g/mL, and cell viability was assessed by cell count after 72-hour exposure. The mean values ± SD of three independent experiments with four replicates in each are shown. Differences were significant (*p* < 0.001) according to *one-way ANOVA*. *Dunnett's multiple comparisons test* was run as *post hoc* test to compare treatment groups with the control group (0, set at 100%); where indicated, differences were statistically significant (^∗∗∗^*p* < 0.001).

We next investigated the RWP toxicity. Primary human monocytes, isolated from peripheral blood of healthy donors, were treated with increasing concentrations of RWP, ranging from 2.5 to 3200 *μ*g/mL. After 72 hours, cell counts were performed. As shown in Figure 1, RWP did not affect the cell number until at a dose of 800 *μ*g/mL. A slight cytotoxicity was observed at the highest tested concentrations of 1600 and 3200 *μ*g/mL, where reductions in the cell number compared with untreated cells (0) were about 20% and 40%, respectively (^∗∗∗^*p* < 0.001, *Dunnett's multiple comparisons test*).

### 3.3. Effect of RWP on the Secretion of IL-1*β*, IL-6, TNF-*α*, and IL-10 Cytokines

To begin to understand the impact of this RWP on the human body, we analyzed its pro- and anti-inflammatory properties. We treated primary human monocytes with LPS, a component of the outer membrane of Gram-negative bacteria that induces inflammatory cascade through the toll-like receptor 4 (TLR4) [27]. LPS leads to the rapid activation of proinflammatory cytokines IL-1*β*, IL-6, and TNF-*α* [37] and the production of IL-10, a potent anti-inflammatory cytokine.

**Figure 2:**
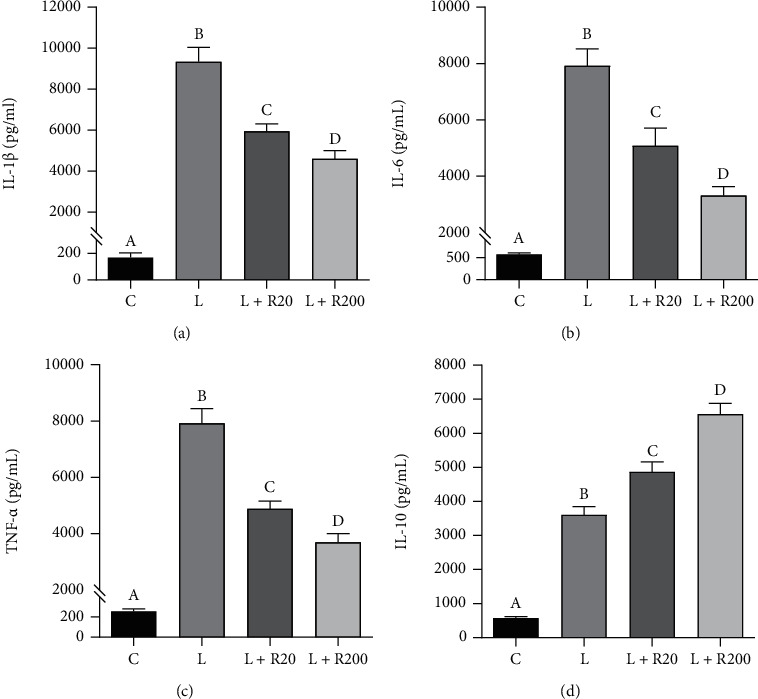
RWP affected the secretion of IL-1*β*, IL-6, TNF-*α*, and IL-10 cytokines. Primary human monocytes were incubated with RWP and activated to macrophages with 1 *μ*g/mL LPS. Twenty-four hours later, the concentrations of the proinflammatory IL-1*β* (a), IL-6 (b), and TNF-*α* (c) and anti-inflammatory IL-10 (d) cytokines in cell culture supernatants were measured. Values represent the means ± SD of three independent experiments with four replicates in each. According to *one-way ANOVA*, differences in IL-1*β* (a), IL-6 (b), TNF-*α* (c), and IL-10 (d) levels were significant (*p* < 0.001). Therefore, *Tukey's post hoc test* was performed, and different letters indicate significant differences between treatments at *p* < 0.05. C: control; L: LPS; R20: RWP 20 *μ*g/mL; R200: RWP 200 *μ*g/mL.

We therefore assessed the release of IL-1*β*, IL-6, TNF-*α*, and IL-10 cytokines after 24 hours of stimulation of monocytes with LPS in the presence or absence of RWP. We observed marked and significant increases in the levels of all the cytokines analyzed after the induction with LPS (Figures 2(a)–2(d): unstimulated cells *vs.* LPS, *p* < 0.001, *Tukey's test*). RWP lowered IL-1*β*, IL-6, and TNF-*α* secretion in a dose-dependent manner (Figures 2(a)–2(c)). Specifically, at a dose of 200 *μ*g/mL, RWP reduced significantly by half the levels of all the proinflammatory cytokines released after stimulation with LPS, whereas RWP at a dose of 20 *μ*g/mL decreased levels of IL-1*β*, IL-6, and TNF-*α* (Figures 2(a)–2(c): LPS *vs.*LPS + RWP 20 *μ*g/mL, p <0.001, *Tukey's test*) by about 35%. On the other hand, IL-10 levels increased significantly in a concentration-dependent manner when monocytes were treated with RWP compared to being triggered only with LPS (Figure 2(d): LPS *vs.*LPS + RWP 20 *μ*g/mL–200 *μ*g/mL, *p* < 0.001, *Tukey's test*).

### 3.4. *Aglianico del Vulture* Red Wine Powder Modulates Expression of Proresolutive Protein AnxA1 in Inflammatory Conditions

**Figure 3:**
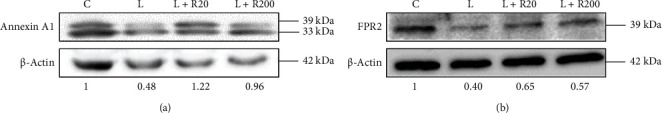
RWP significantly restored expression of AnxA1 in LPS-induced inflammation. Primary human monocytes were incubated with RWP 20 *μ*g/mL or RWP 200 *μ*g/mL and activated with 1 *μ*g/mL LPS. Expression of AnxA1 (a) and FPR2 (b) was assessed following 24 h treatment with LPS. Protein expression was quantified by using optical density (O.D.) ratio for AnxA1 or FPR2 versus *β*-actin; normalized values obtained are reported under western blot images. C: control; L: LPS; R20: RWP 20 *μ*g/mL; R200: RWP 200 *μ*g/mL.

We next focused on the role of the AnxA1/FPR2 axis in LPS-induced inflammation *in vitro*. AnxA1 is a proresolutive protein induced and activated during inflammation, aimed at limiting tissue damage and restoring homeostasis through activation of Formyl Peptide Receptor 2 (FPR2) [3]. LPS treatment reduced total AnxA1 expression, while pretreatment with RWP, at both concentration used (20 or 200 *μ*g/mL), was able to restore its physiological amount, thus preventing cells to undergo excessive inflammation (Figure 3(a)). In addition, expression of FPR2 receptor was downregulated by LPS administration (Figure 3(b)). Although not significantly, RWP slightly increased FPR2 expression, only causing a positive trend (Figure 3(b)).

### 3.5. Effect on Expression, Promoter Activity and Nuclear Translocation of NF-*κ*B

When LPS binds to TLR4 on the surface of macrophages, a signal transduction cascade leading to transcription of specific enzymes, such as COX2, matrix metalloproteinase-9, and inflammatory cytokines TNF-*α*, IL-1, IL-6, IL-8, and chemokines [38]. The NF-*κ*B pathway plays a central role for the protective properties of a moderate wine consumption [39]. Therefore, we evaluated if the red wine powder from *Aglianico del Vulture* will affect the NF-*κ*B pathway. We focused on the expression of NF-*κ*B, its promoter activity, and its cellular localization.

**Figure 4:**
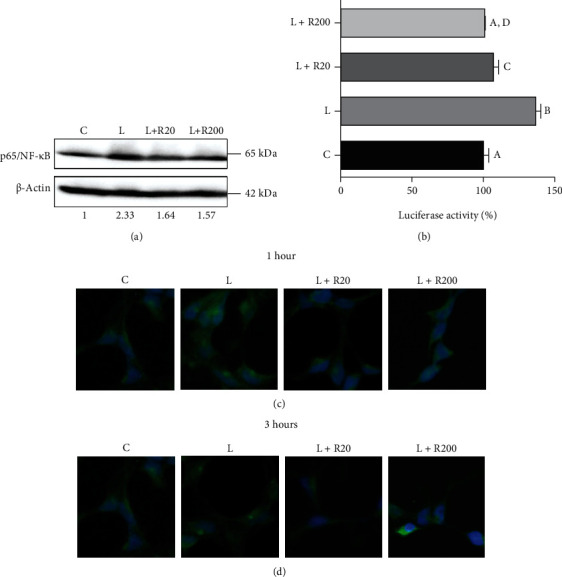
RWP inhibited NF-*κ*B transcription factor. (a) Primary human monocytes were incubated with RWP 20 *μ*g/mL or RWP 200 *μ*g/mL and activated to macrophages with 1 *μ*g/mL LPS. Specific antibodies detected the expression levels of subunit p65 of NF-*κ*B and *β*-actin. The intensities of immunolabeled protein bands were measured by a quantitative software and normalized to *β*-actin; values obtained are reported under western blot images. Protein expression levels in control sample were taken as 1, and other samples were expressed in proportion to the control. (b) HEK293 cells were transfected with NF-*κ*B luciferase reporter plasmid and treated with LPS in the presence or not of RWP 20 *μ*g/mL or RWP 200 *μ*g/mL. Bar chart reports the mean values ± SD of three independent experiments, each in triplicate. According to *one-way ANOVA*, differences were significant (*p* < 0.001). Therefore, *Tukey's post hoc test* was performed, and different letters indicate significant differences between treatments at *p* < 0.05. (c-d) Immunocytochemistry experiments were performed to identify the cellular localization of subunit p65 of NF-*κ*B, recognized by a specific antibody. Cells were treated with RWP 20 *μ*g/mL or RWP 200 *μ*g/mL and activated with LPS. C: control; L: LPS; R20: RWP 20 *μ*g/mL; R200: RWP 200 *μ*g/mL.

For this, primary human monocytes were treated with LPS in the presence or absence of RWP. LPS induced a marked overexpression of subunit p65 of NF-*κ*B (Figure 4(a)). RWP, used as both 20 *μ*g/mL and 200 *μ*g/mL, reduced p65/NF-*κ*B protein levels to about 40% (Figure 4(a)). Likewise, even if significant (*Tukey's test*), no strong differences were found in the ability to inhibit NF-*κ*B promoter activity between RWP 20 *μ*g/mL and RWP 200 *μ*g/mL (Figure 4(b)). To monitor the effect of RWP on the NF-*κ*B signal transduction pathway, HEK293 cells were transiently transfected with a NF-*κ*B reporter plasmid containing a firefly luciferase gene driven by five copies of NF-*κ*B response element located upstream of the minimal TATA box promoter. After activation by proinflammatory stimuli, endogenous NF-*κ*B binds to the DNA response elements, inducing transcription of the luciferase reporter gene. When cells were treated with LPS, we observed a significant increase in luciferase activity, when compared to untreated cells (Figure 4(b) C *vs.* LPS, *p* < 0.001, *Tukey's test*); RWP reported the levels of luciferase activity in LPS-triggered cells at values similar to those of control. Next, we analyzed the cellular localization of p65, the subunit of NF-*κ*B. After 1 hour (Figure 4(c)) and 3 hours (Figure 4(d)) of treatment with LPS, we observed the translocation of subunit p65 of NF-*κ*B from the cytosol to the nucleus. In cells cotreated with RWP, the main NF-*κ*B localization was cytosolic (Figures 4(c) and 4(d)). Altogether, these data suggest that RWP plays a role in inhibiting the NF-*κ*B pathway.

### 3.6. Effect on the Citrate Export Pathway: Focus on CIC and Citrate

Among the proinflammatory genes activated by NF-*κ*B is *SLC25A1*, which encodes the mitochondrial citrate carrier (CIC). It is the component of the citrate pathway responsible for the export of the citrate withdrawn from the Krebs cycle to the cytosol after LPS stimulation [13, 14].

**Figure 5:**
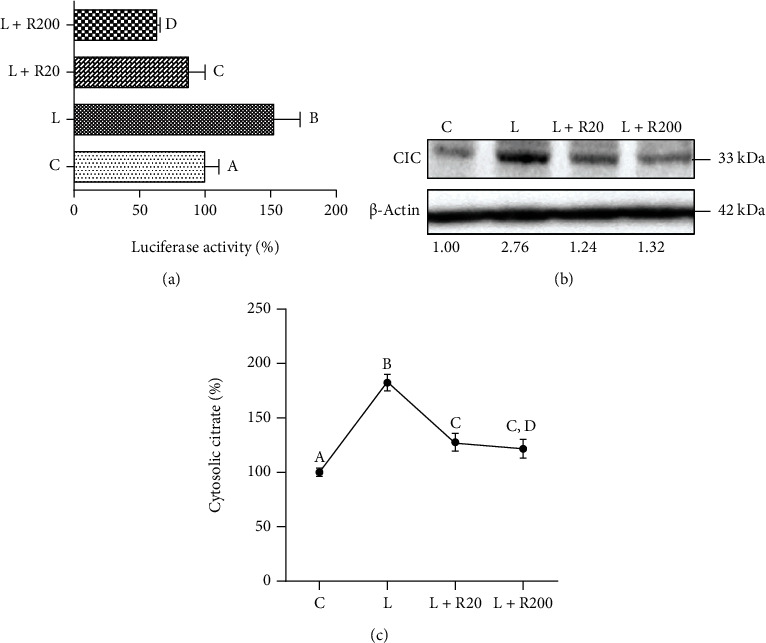
Effect of RWP on CIC and cytosolic citrate. (a) HEK293 cells were transiently transfected with SLC25A1pGL3, the pGL3 basic-LUC vector encompassing the −1785/−20 bp region of the *SLC25A1* gene cloned upstream of the luciferase reporter gene. Then, cells were triggered with LPS in the absence (LPS) or in the presence of RWP 20 *μ*g/mL or RWP 200 *μ*g/mL. Unstimulated cells (c) were used as a negative control. The luciferase gene reporter activity was assessed after 24 hours. (b) Primary human monocytes, preincubated for 1 hour with RWP 20 *μ*g/mL or RWP 200 *μ*g/mL, were activated to macrophages with LPS, and CIC protein levels were evaluated. CIC and *β*-actin proteins were immunodecorated with specific antibodies. The intensities of immunolabeled protein bands were measured by using a quantitative software and normalized to *β*-actin: values obtained are reported under western blot images. Protein expression levels in control sample were taken as 1, and other samples were expressed in proportion of the control. (c) In cells treated as in (b), cytosolic citrate levels were quantified. In (a) and (c), values represent means ± SD of three experiments with three replicates in each. Statistical analysis was performed by o*ne-way ANOVA* followed by *Tukey's test* for multiple comparisons. Different letters indicate significant differences at *p* < 0.05. C: control; L: LPS; R20: RWP 20 *μ*g/mL; R200: RWP 200 *μ*g/mL.

The human *SLC25A1* promoter contains two NF-*κ*B response elements at positions −414/−405 bp and −1314/−1305 bp. To verify if RWP induced alterations in the transcription rate of the *SLC25A1* promoter, we transfected HEK293 cells with the previously described SLC25A1pGL3—a vector with *SLC25A1*—promoter encompassing the −1785/−20 bp region of the *SLC25A1* gene cloned upstream of the luciferase reporter gene [29]. Next, we treated with LPS, in the presence or absence of RWP. We observed that RWP significantly reduced luciferase activity in a dose-dependent manner by 50% (RWP 20 *μ*g/mL) and 60% (RWP 200 *μ*g/mL) in cells upon LPS stimulation (*Tukey's test*, Figure 5(a)). We also measured the protein levels of CIC and confirmed a downregulation at the protein level (Figure 5(b)). However, we did not observe a dose-dependent effect at the protein level. LPS induced a threefold overexpression of CIC with respect to untreated cells (Figure 5(b)), and RWP, as both 20 *μ*g/mL and 200 *μ*g/mL, reported CIC levels to values similar to control cells (Figure 5(b)). A correspondent lowering in cytosolic citrate levels was detected: RWP either as 20 *μ*g/mL or as 200 *μ*g/mL reduced cytosolic citrate by around 40% with respect to LPS-triggered cells (Figure 5(c)).

### 3.7. Effect on the Citrate Export Pathway: Focus on ATP Citrate Lyase

**Figure 6:**
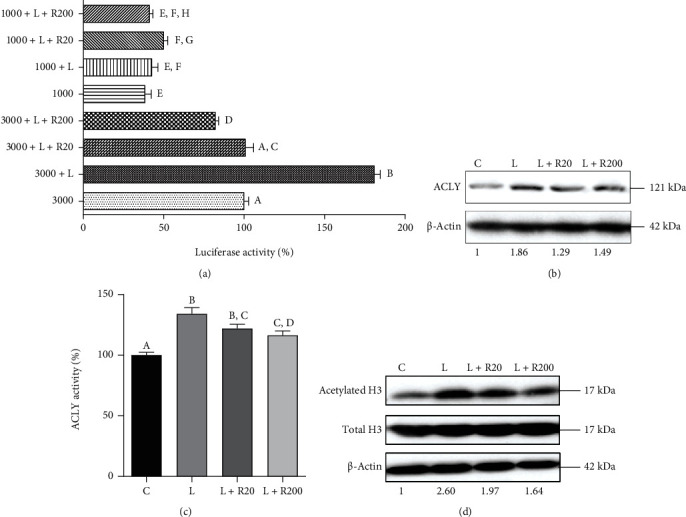
Effect of RWP on ACLY. (a) HEK293 cells were transiently transfected with pGL3 basic-LUC vectors containing the −3116/−20 bp full-length region of the *ACLY* gene promoter (3000) or a truncated version of this region (1000). Then, cells were triggered with LPS in the absence (LPS) or in the presence of RWP 20 *μ*g/mL or RWP 200 *μ*g/mL. Unstimulated cells were used as a negative control. The luciferase gene reporter activity was assessed after 24 hours. Primary human monocytes, preincubated for 1 hour with RWP, were activated to macrophages with LPS, and protein levels of ACLY (b) and acetylated H3 and total H3 (d) were evaluated. In (b, d) ACLY, acetylated H3, total H3, and *β*-actin proteins were immunodecorated with specific antibodies. The intensities of immunolabeled protein bands were measured by using a quantitative software and normalized to *β*-actin: values obtained are reported under western blot images. Protein expression levels in control sample were taken as 1, and other samples were expressed in the proportion of the control. (c) In cells treated as in (b, d) ACLY enzymatic activity was quantified. In (a) and (c), values represent means ± SD of three experiments with three replicates in each. Statistical analysis was performed by o*ne-way ANOVA* followed by *Tukey's test* for multiple comparisons. Different letters indicate significant differences at *p* < 0.05. C: control; L: LPS; R20: RWP 20 *μ*g/mL; R200: RWP 200 *μ*g/mL.

ACLY is upregulated very early in macrophages activated by LPS or by TNF-*α* and/or interferon *γ* (IFN*γ*) as well as in inflammatory conditions [15, 19, 20]. To determine whether RWP affected NF-*κ*B binding to ACLY, we analyzed the promoter region of the human *ACLY* gene. Since it contains an active NF-*κ*B response element localized at −2048/−2038 bp [15], cells were transiently transfected with pGL3 basic-LUC vectors containing the −3116/−20 bp full-length region of the *ACLY* gene promoter (called “3000,” Figure 6(a)), including the NF-*κ*B response element. As a control, we also transfected cells with a mutant version, containing a truncated version of this region (called “1000,” Figure 6(a)), without the NF-*κ*B response element. Both plasmids were then used to test the effect of RWP on luciferase activity. The absence of the binding site for NF-*κ*B was responsible for the lower *ACLY* gene promoter activity in cells transfected with 1000 than 3000 (Figure 6(a)). Following 24 hours of LPS treatment, the strongest promoter activity was registered in 3000 transfected cells (3000 + LPS, Figure 6(a)). The luciferase gene reporter activity was significantly reduced to levels similar to unstimulated cells (3000, Figure 6(a)) by RWP 20 *μ*g/mL and to even lower levels by RWP 200 *μ*g/mL (Figure 6(a)). RWP induced a parallel decrease in ACLY protein levels and enzymatic activity in LPS-triggered macrophages (Figures 6(b) and 6(c)). More in detail, LPS induced a twofold increase in ACLY expression levels (Figure 6(b)) and a 35% rise in ACLY activity (Figure 6(c)). No significant differences were observed between RWP 20 *μ*g/mL and 200 *μ*g/mL in bringing ACLY protein levels (Figure 6(b)) and activity (Figure 6(c)) down. These results, together with CIC and cytosolic citrate depletion, define a crucial role of RWP in immunometabolism.

Moreover, it has been recently demonstrated that rapid metabolic changes in LPS-induced macrophages are important to increase ACLY-derived acetyl-CoA that in turn leads to histone acetylation [40, 41], critical in regulating global chromatin accessibility and gene transcription. Transcriptional regulation of genes involved in macrophage activation and inactivation or determination of their polarization state occurs through histone modifications [42]. Therefore, changes in histone acetylation have a great impact in inflammation. We show that after LPS stimulation the levels of ACLY went up, with consequences in increased H3 histone acetylation (Figure 6(d)). On the other hand, treatment of cells with RWP lowered acetylated H3 in a dose-dependent manner (Figure 6(d)), suggesting an epigenetic activity of RWP.

### 3.8. Effect on the Levels of Inflammatory Mediators Downstream the Citrate Pathway: ROS and NO^·^

**Figure 7:**
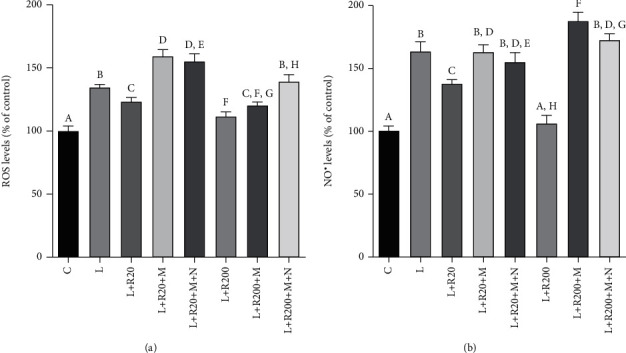
RWP lowered ROS and NO^·^ levels, restored by addition of exogenous malate and NADPH. Primary human monocytes were treated with LPS (L) in absence or in presence of RWP alone or plus malate and NADPH. Following 24 hours, ROS (a) and NO^·^ (b) levels were evaluated and expressed as the percentage of unstimulated cells (set at 100%). Mean values ± SD of three replicate independent experiments with five replicates in each are shown. According to *one-way* ANOVA, differences in ROS (a) and NO^·^ (b) levels were significant (*p* < 0.001). Therefore, *Tukey's post hoc test* was performed, and different letters indicate significant differences between treatments at *p* < 0.05. C: control; L: LPS; R20: RWP 20 *μ*g/mL; R200: RWP 200 *μ*g/mL; M: malate; N: NADPH.

Since RWP downregulated CIC and ACLY, we analyzed its role in regulating the citrate pathway in LPS-activated macrophages. Citrate cleavage made by ACLY supplies intermediaries for the biosynthesis of three inflammatory mediators: ROS, NO^·^, and prostaglandin E_2_ [16]. In LPS-triggered macrophages, the accumulated citrate is exported by CIC from the mitochondria to the cytosol and converted by ACLY into oxaloacetate and acetyl-CoA. OAA is converted to pyruvate with consequent production of NADPH [43], used for ROS and NO^·^ synthesis. Our analysis showed enhanced and significant releases of ROS and NO^·^ when human primary monocytes were treated with LPS (Figures 7(a) and 7(b)). RWP, on the other hand, reduced the levels of reactive oxygen species and nitric oxide in a dose-dependent manner (Figures 7(a) and 7(b)). In particular, RWP 20 *μ*g/mL decreased by 10 and 15% the levels of ROS and NO^·^, respectively, with respect to cells treated only with LPS; reductions induced by RWP 200 *μ*g/mL were about 20 and 35% (Figures 7(a) and 7(b)).

Malate and NADPH are two metabolites downstream the citrate pathway: in particular, malate is produced in the reaction catalyzed by MDH1, while NADPH derived from the ME1 cleavage of malate in pyruvate. Exogenous malate used alone or in combination with NADPH reverts ACLY inhibition phenotype leading to a huge increase of both ROS and NO^·^ inflammatory mediators. As shown in Figures 6(a) and 6(b), the addition of malate alone or in combination with NADPH was sufficient to increase ROS as well as NO^·^ levels in LPS-triggered cells treated with RWP. Therefore, the effect of RWP on ROS and NO^·^ levels could occur through the citrate pathway suppression together with a direct inhibition of NF-*κ*B, which controls the expression of ACLY, but also the expression of *NADPH oxidase* and *iNOS* genes [44, 45].

### 3.9. Inhibition of COX2 and Reduction of PGE_2_ Level: Involvement of the Citrate Pathway

**Figure 8:**
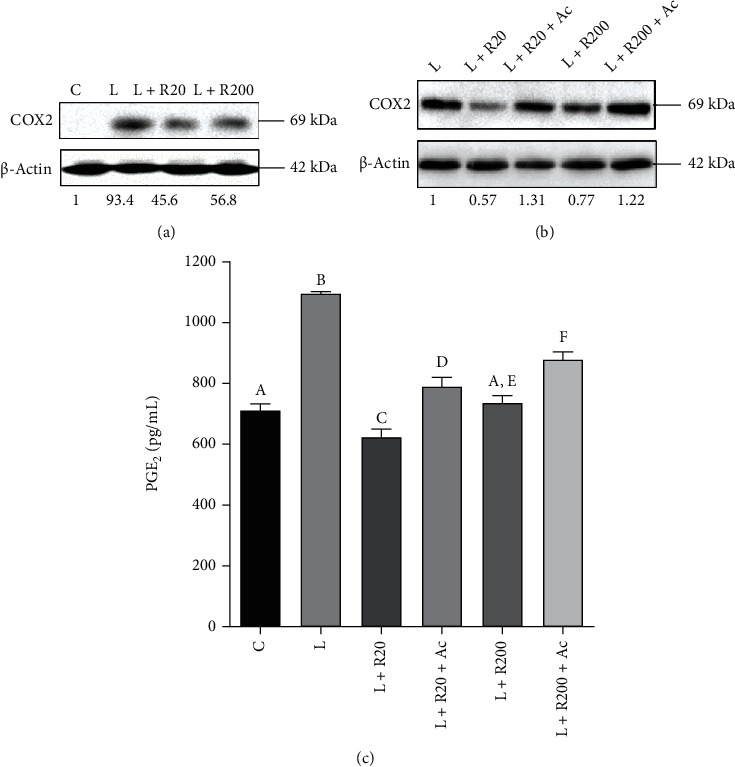
RWP lowered COX2 and PGE_2_ levels. Primary human monocytes were activated to macrophages with LPS (LPS) in the absence or in the presence of RWP alone or plus acetate and COX2 (a, b), and PGE_2_ levels were quantified. In (a) and (b), COX2 and *β*-actin proteins were immunodecorated with specific antibodies. The intensities of immunolabeled protein bands were measured by using a quantitative software and normalized to *β*-actin; values obtained are reported under western blot images. Protein expression levels in control sample were taken as 1, and other samples were expressed in proportion to the control. (c) Following 48 hours, PGE_2_ levels were evaluated and expressed as the percentage of the levels in untreated cells (set at 100%). Mean values ± SD of three replicate independent experiments with three replicates in each are shown. According to *one-way* ANOVA, differences were significant (*p* < 0.001). Therefore, *Tukey's post hoc test* was performed, and different letters indicate significant differences between treatments at *p* < 0.05. C: control; L: LPS; R20: RWP 20 *μ*g/mL; R200: RWP 200 *μ*g/mL; Ac: acetate.

Finally, the focus was set on the other inflammatory mediator downstream the citrate pathway, PGE_2_, and on the enzyme COX2 responsible for its synthesis. It is well known that wine polyphenols inhibit COX2 in inflammation induced by LPS. *Aglianico del Vulture* red wine powder at 20 *μ*g/mL as well as 200 *μ*g/mL reduced COX2 expression levels almost the half with respect to macrophages activated only with LPS (Figure 8(a)). The strongest reduction was observed with the lowest tested concentration of RWP (Figure 8(a)). Interestingly, acetate, a metabolite downstream of the citrate pathway, reverted the inhibition of COX2 induced by RWP (Figure 8(b)). A similar trend was observed when PGE_2_ levels were measured in cell culture supernatants after 48 hours of incubation with LPS (Figure 8(c)). In details, RWP-stimulated cells showed PGE_2_ levels similar to unstimulated cells. We observed a 40% decrease in PGE_2_ levels when compared to LPS-triggered cells. On the other hand, the addition of acetate brought PGE_2_ levels up (Figure 8(c)). This decreased PGE_2_ level is most likely because of a decrease in PGE_2_ production, due to a reduced availability of precursors for PGE_2_ synthesis: as acetate can be converted to acetyl-CoA by acetyl-CoA synthase (ACSS), adding exogenous acetate rescues the effect of ACLY inhibition on PGE_2_ production. These data, with the previous regarding the effect of RWP on ROS and NO^·^, strengthened and confirmed our hypothesis that the citrate pathway is a target of RWP in carrying out its anti-inflammatory activity.

## 4. Discussion

In this study, for the first time, we have investigated the biological properties of *Aglianico del Vulture* red wine and we have shown that it exerts potential health benefits thanks to its content in polyphenols well known to act as immunomodulators and anti-inflammatory molecules [1, 2, 5, 6, 9, 10, 46–49].

Malvidin 3-*O*-glucoside and cyanidin-3-*O*-glucoside are the most abundant phenolic compounds we have found, in accordance with the typical anthocyanin profiling of *Aglianico* wine, in which malvidin 3-*O*-glucoside represents about 60% while cyanidin-3-*O*-glucoside and delphinidin-3-*O*-glucoside are around 5% of total anthocyanidins [34]. These compounds were present in higher concentration with respect to another DOC red wine *Carignano del Sulcis*, cultivated in the southwestern region of Sardinia (Italy) [24]. In our sample, the concentration of the stilbene resveratrol was lower (0.053 ± 0.01 mg/100 mL) than that of red wines from Veneto region (Italy), in which resveratrol averaged 0.083 mg/100 mL [50], and Campania region (Italy) [26]. On the other hand, the flavonol quercetin was more abundant in *Aglianico del Vulture* red wine, compared to the last red wines from Campania, in particular with respect to *Aglianico del Benevento* [26]. Finally, among the phenolic acids, a higher rate of caffeic acid was found in our sample in comparison with wines counted among the best wines for anti-inflammatory properties for their abundance in phenolic compounds, such as Cabernet Sauvignon, Merlot, Syrah, and Carménère [47].

Human primary monocytes have been used for our investigations. Cells were treated with LPS, which triggers innate immune responses leading to the secretion of cytokines IL-1*β*, IL-6, and TNF-*α* and proinflammatory mediators blocked by *Aglianico del Vulture* powder. On the other hand, RWP induced an increased release of IL-10, necessary to initiate host defence against microbial invasion [37]. However, excessive secretion of proinflammatory cytokines could be deleterious for the host since they cause systemic metabolic and hemodynamic disturbances. For that reason, macrophages produce IL-10, a potent anti-inflammatory cytokine produced by macrophages as a negative-feedback mechanism to dampen excessive inflammation during infection.

These data are in line with the rescuing effect exerted by RWP on AnxA1 levels, which were decreased upon LPS activation. This impairment could lead to uncontrolled inflammation resulting in chronic disease following unbalance between inflammation and resolution [51]. Thus, RWP could modulate inflammatory response with an alternate mechanism, which controls the resolution pathway associated to the AnxA1/FPR2 axis.

In vitro and in vivo studies reported that polyphenols contained in red grapes and red wines are able to abrogate the LPS-mediated activation of NF-*κ*B with consequent attenuation of the storm of proinflammatory cytokines released by monocytes [52], so that the NF-*κ*B pathway has been identified as a critical target for the protective properties of a moderate wine consumption. Therefore, our attention was directed to evaluate the effect of RWP on an NF-*κ*B transcription factor. As was to be expected, RWP reduced the expression of p65 subunit of NF-*κ*B, promoter activity, and nuclear translocation of NF-*κ*B. As a consequence of NF-*κ*B inhibition, *SLC25A1* and *ACLY* gene promoter activities lowered with consequent reduction in CIC and ACLY protein levels; a parallel decrease in cytosolic citrate concentration and inflammatory mediators linked to the citrate pathway (ROS, NO^·^, and PGE_2_) was observed. Obviously, the effect of the tested powder on ROS, NO^·^, and PGE_2_ could also be a consequence of the direct inhibition of NF-*κ*B since under its transcriptional controls are genes encoding for iNOS and COX2. However, the inhibition of the citrate pathway has a central role. In fact, treatments with metabolites downstream the citrate pathway removed RWP inhibitory effects on proinflammatory mediators: exogenous malate alone or in combination with NADPH reverted the reduction of ROS and NO^·^ levels; acetate did the same on PGE_2_ concentration and COX2 expression levels. Analogous involvement of the citrate pathway was found in Down syndrome, where hydroxycitrate—a natural ACLY inhibitor—reduced the typical prooxidant status, but the addition of malate or NADPH abolished its antioxidant effect [19]. Similarly, *Pistacia lentiscus* hydrosol exhibited its anti-inflammatory activity acting through the citrate pathway [30].

Interestingly, RWP exerts its effect also at the epigenetic level, as shown by reduction of the acetylation of H3 histone. Acetyl-CoA, a product of the citrate pathway needed for histone acetylation [17], represents a key node in metabolism due to its intersection with many metabolic pathways and transformations, influencing the regulation of numerous life processes.

In addition to the effect on the citrate pathway, it cannot be ruled out that the RWP compounds contained in this wine might have other beneficial effect. In fact, it is known that increasing of cytokines IL-1*β* and TNF-*α*, with subsequent increased expression of adhesion molecules, contributes to lipid accumulation within the atheroma and dysregulated activity of vascular smooth muscle cells [53]. Thus, reduction of proinflammatory cytokines by RWP might also positively affect the cardiovascular system. Furthermore, since inflammation and ROS may induce the increase/decrease of several miRNAs, including oxidative stress-responsive miRNAs [54], it could also be hypothesized that these phytochemicals may also control the expression of some target miRNAs in both normal and pathological conditions [55]. For example, the phytochemical epigallocatechin gallate may act as an epigenetic modulator of DNA methylation and chromatin remodeling, leading to the alteration of gene expression and modification of miRNA activities [56]. Other beneficial effects, such as glucose homeostasis, mitochondrial function, energy metabolism, and stress responses, have been ascribed to phenolic compounds [57]. However, further investigations are needed to elucidate these and other potential beneficial effects of RWP.

In conclusion, our study highlights for the first time the contribution of red wine *Aglianico del Vulture* phenols in modulation of inflammatory response. Notably, our findings suggest a specific signature of this red wine showing its own phenolic profile. The underlying mechanism is associated to different pathways, including the suppression of inflammatory mediators and the inhibition of NF-*κ*B and the citrate pathway. Bioactive compounds from red wines such as malvidin 3-*O*-glucoside and cyanidin-3-*O*-glucoside, quercetin and resveratrol, have been shown to inhibit inflammatory mediators via NF-*κ*B [46, 48, 49]. The involvement of the citrate pathway is the strongest novelty, since this has never been investigated so far as a possible mechanism of action for any wine compounds. Here, we demonstrate that this pathway mediates several anti-inflammatory effects of the red wine *Aglianico del Vulture* phenols. In the recent years, the activation of ACLY and CIC—constituents of the citrate pathway—has been linked to the presence of inflammatory conditions [46, 48, 49]. Therefore, the citrate pathway seems a new hopeful target of inflammation. In this context, its inhibition by red wine *Aglianico del Vulture* phenols—as a molecular mechanism underlying the regulation of macrophage function—could reveal very interesting applications in the prevention and treatment of inflammatory chronic diseases simply through bioactive food compounds.

## 5. Conclusions

For the first time, this study investigates the immunomodulatory and anti-inflammatory potential of red wine powder (RWP) extracted from the Italian red wine *Aglianico del Vulture.* RWP reduces IL-1*β*, IL-6, and TNF-*α* proinflammatory while increasing IL-10 anti-inflammatory cytokine secretion and inhibiting NF-*κ*B promoter activity in macrophages induced by LPS. In addition, RWP activates proresolutive pathways by restoring Annexin A1 levels. Beyond the classical targets of macrophage function, we also identify the citrate pathway as a RWP target in carrying out its anti-inflammatory activity since, by reducing CIC and ACLY protein levels, ACLY enzymatic activity, RWP lowers ROS, NO^·^, PGE_2_, and histone acetylation levels. Overall findings evidence that *Aglianico del Vulture* powder suppresses inflammatory pathways and activates proresolutive processes hinting the potential value of RWP in the prevention and treatment of inflammatory conditions as well as inflammatory chronic diseases.

## Figures and Tables

**Table 1:** Composition of the red wine powder (RWP) obtained from Aglianico del Vulture (harvest 2018). Mass spectrometry-based analyses were carried out to evaluate the amount of specialized metabolites in RWP. The mean values ± standard deviation (SD) from at least three independent experiments, each in triplicate, are reported.

	mg/100 mL ± SD
*Phenolic acids*	
Caffeic acid	0.218 ± 0.047
Coumaric acid	0.078 ± 0.002
*Stilbenes*	
Resveratrol	0.053 ± 0.01
*Anthocyanidins*	
Delphinidin-3-O-glucoside	0.072 ± 0.003
Cyanidin-3-O-glucoside	1.30 ± 0.18
Malvidin-3-O-glucoside	14.00 ± 0.23
*Flavonols*	
Quercetin	0.785 ± 0.02

## Data Availability

The data used to support the findings of this study are available from the corresponding author upon request.

## Supplementary Materials

Supplementary Materials Figure S1: MRM data in both positive and negative ions of a mix standard solution.
