# Supplementary material for: Phenolic Compounds of Red Wine Aglianico del Vulture Modulate the Functional Activity of Macrophages via Inhibition of NF-κB and the Citrate Pathway
Source: Oxid Med Cell Longev. 2021 May 25;2021:5533793. doi: 10.1155/2021/5533793 (PMC8172326; doi:10.1155/2021/5533793)
Supplement: Supplementary Materials — Figure S1: MRM data in both positive and negative ions of a mix standard solution. [file 5533793.f1.docx]

**Supplementary Figure**


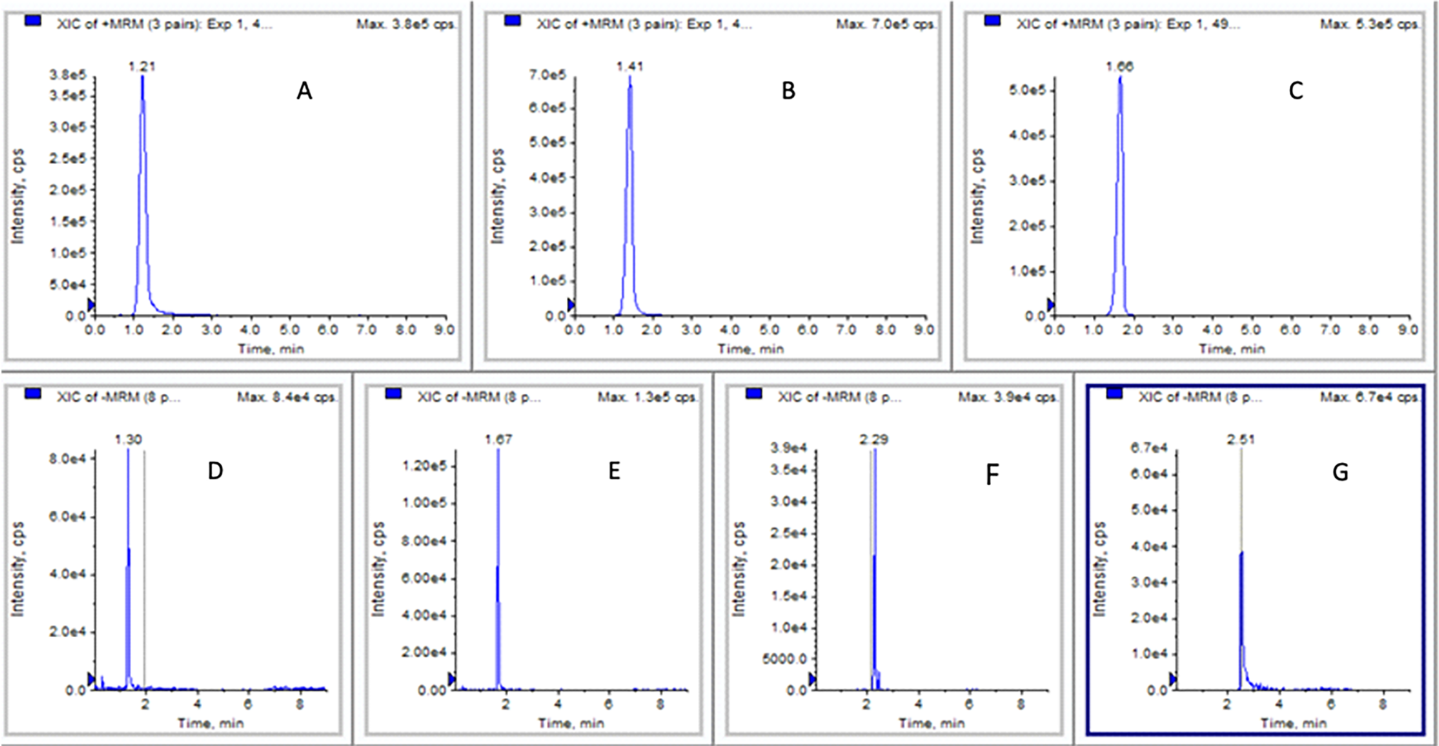


**Figure S1. MRM data in both positive and negative ions of a mix standard solution** A: delphinidin 3-*O*-glucoside; B: cyanidin 3-*O*-glucoside; C: malvidin 3-*O*-glucoside; D: caffeic acid; E: coumaric acid; F: resveratrol and G: quercetin.
